# Noninvasive Seizure Onset Zone Localization Using Janashia–Lagvilava Algorithm-Based Spectral Factorization in Granger Causality

**DOI:** 10.3390/brainsci15121334

**Published:** 2025-12-15

**Authors:** Sofia Kasradze, Giorgi Lomidze, Lasha Ephremidze, Tamar Gagoshidze, Giorgi Japaridze, Maia Alkhidze, Tamar Jishkariani, Mukesh Dhamala

**Affiliations:** 1 Institute of Neurology and Neuropsychology, 83/11 Vazha-Pshavela Ave., 0186 Tbilisi, Georgia; sofiakas@gmail.com (S.K.); gjaparidze58@gmail.com (G.J.); maia_alkhidze@yahoo.com (M.A.); tatuka80@yahoo.com (T.J.); 2 Faculty of Medicine, Caucasus International University, 73 Chargali Str., 0141 Tbilisi, Georgia; 3 Faculty of Medicine, European University, 17 D. Sarajishvili Ave., 0189 Tbilisi, Georgia; 4 School of Mathematics, Kutaisi International University, Akhalgazrdoba Ave. Lane 5/7, 4600 Kutaisi, Georgia; lasha.ephremidze@kiu.edu.ge; 5 Faculty of Psychology and Educational Sciences, I. Javakhisvili Tbilisi State University, 1 Ilia Tchavtchavadze Avenue, 0179 Tbilisi, Georgia; tamar.gagoshidze@tsu.ge; 6 Neuroscience Institute, Physics and Astronomy, Math & Statistics, Georgia State University, Atlanta, GA 30302, USA; mdhamala@gsu.edu

**Keywords:** drug-resistant epilepsy, seizure onset zone, noninvasive SOZ localization, matrix spectral factorization, Janashia–Lagvilava algorithm, Granger causality

## Abstract

**Background/Objectives:** Precise identification of seizure onset zones (SOZs) and their propagation pathways is essential for effective epilepsy surgery and other interventional therapies and is typically achieved through invasive electrophysiological recordings such as intracranial electroencephalography (EEG). Previous research has demonstrated that analyzing information flow patterns, particularly in high-frequency oscillations (>80 Hz) using parametric and Wilson algorithm (WL)-based nonparametric Granger causality (GC), is valuable for SOZ identification. In this study, we analyzed scalp EEG recordings from epilepsy patients using an alternative nonparametric GC approach based on spectral density matrix factorization via the Janashia–Lagvilava algorithm (JLA). The aim of this study is to evaluate the effectiveness of JLA-based matrix factorization in nonparametric GC for noninvasively identifying seizure onset zones from ictal EEG recordings in patients with drug-resistant epilepsy. **Methods:** Two regions of interest (ROIs) in pairs were isolated across different time epochs in six patients referred for presurgical evaluation. To apply the nonparametric Granger causality (GC) estimation approach to the EEG recordings from these regions, the cross-power spectral density matrix was first computed using the multitaper method and subsequently factorized using the JLA. This factorization yielded the transfer function and noise covariance matrix required for GC estimation. GC values were then obtained at different prediction time steps (measured in milliseconds). These estimates were used to confirm the visually suspected seizure onset regions and their propagation pathways. **Results:** JLA-based spectral factorization applied within the Granger causality framework successfully identified SOZs and their propagation patterns from scalp EEG recordings, demonstrating alignment with positive surgical outcomes (Engel Class I) in all six cases. **Conclusions:** JLA-based spectral factorization in nonparametric Granger causality shows strong potential not only for accurate SOZ localization to support diagnosis and treatment, but also for broader applications in uncovering information flow patterns in neuroimaging and computational neuroscience.

## 1. Introduction

Epilepsy is one of the most common multi-etiologic, noncommunicable, chronic neurological disorders of the brain [1], affecting approximately 50 million people worldwide [2]. It is characterized by recurrent epileptic seizures and typically requires long-term anti-seizure drug (ASD) treatment. Complete seizure control can be achieved in up to 65–70% of patients with appropriate ASD therapy; however, in the remaining 30–35%, seizures remain difficult to control even with correct pharmacological management. These cases are classified as drug-resistant epilepsy (DRE) [3], for which epilepsy surgery is considered the most effective alternative to achieve seizure control. Nevertheless, surgical treatment is an option only for eligible candidates with DRE, and after presurgical evaluation, only about one quarter of these patients ultimately qualify for surgery [3].

The success of epilepsy surgery depends on the early identification of appropriate candidates and on selecting those most likely to achieve postoperative seizure freedom [4]. During the initial phase of presurgical evaluation, patients undergo detailed, multidisciplinary assessment according to a noninvasive protocol that includes long-term video-EEG monitoring, multimodal neuroimaging, and neuropsychological testing [5]. The central objective of this evaluation in DRE is to accurately localize the seizure onset zone (SOZ) for potential surgical resection and to characterize the patterns of seizure propagation. Among these modalities, video-EEG monitoring and expert interpretation of ictal and interictal EEG patterns by a qualified clinical neurophysiologist remain essential.

However, 10–40% of patients who undergo presurgical evaluation do not exhibit clearly localizable seizures using scalp EEG, multimodal imaging, or MEG, and many of these individuals subsequently require invasive intracranial EEG (IEEG) recordings with grid or depth electrodes [6]. Despite recent trends toward increasing the number of implanted electrodes—often exceeding 100 per patient—greater electrode density has not always translated into improved diagnostic precision. Sampling of the SOZ may remain incomplete, and traditional electrographic criteria for determining seizure onset on IEEG may be insufficient in a substantial proportion of cases [6]. Consequently, no single diagnostic tool can unambiguously delineate the SOZ, and clinicians must rely on the integration of multiple complementary modalities.

Recent studies suggest that high-frequency neural oscillations (HFOs), including “ripples” (<250 Hz) [7] and “fast ripples” (>250 Hz), are reliable biomarkers for early detection of epileptic foci and seizure activity in the brain [8,9,10,11]. These rapid oscillations are considered the most sensitive electrophysiological markers for localizing the epileptic SOZ, and may be more specific than spikes, particularly in MRI-negative cases [12,13]. One of the most advanced approaches evaluating such interactions is the Geweke’s formulation of Granger causality (GC) [14].

Granger causality (GC) is a statistical method used to assess directional influences between simultaneously recorded time series and involves matrix spectral factorization. Evidence suggests that GC can be useful for localizing seizure dipoles [15]. To date, Wilson's algorithm (WA) for matrix factorization [16] has been the most widely applied approach for the nonparametric GC [17].

Another approach is the matrix spectral factorization method developed by Janashia and Lagvilava in 1999, based on a long mathematical tradition in Georgia. Unlike WA, this method is built on a different mathematical framework that exploits a specific class of unitary matrix functions. It is known as the Janashia–Lagvilava matrix spectral factorization algorithm (JLA) [18]. Although the JLA has recently attracted attention in mathematical and engineering research [19,20], its potential use in clinical neurophysiology has not been explored.

In the present study, we apply a nonparametric GC approach based on JLA spectral density matrix factorization to scalp EEG recordings from patients with drug-resistant epilepsy, in order to demonstrate its feasibility as a possible tool for SOZ assessment.

The aim of our study was to determine the SOZ and trajectory of its propagation by the GC using the JLA method and to evaluate the possibilities of the JLA method in clinical practice.

## 2. Methods

This ongoing cohort study is being conducted at the Epilepsy Prevention and Control Center of the Institute of Neurology and Neuropsychology (Tbilisi, Georgia). In the first stage, we analyzed preliminary data from six adult patients with drug-resistant epilepsy (DRE) who underwent presurgical evaluation and were selected as candidates for epilepsy surgery. In all cases, an interdisciplinary assessment was performed using a noninvasive presurgical protocol (phase 1a) [5].

### 2.1. Seizure Semiology

Seizure semiology was classified according to the 2017 ILAE seizure classification system [1]. All study participants presented with focal seizures.

### 2.2. MRI

3T MRI scans were obtained in all cases using an epilepsy-specific protocol. To improve sensitivity and specificity in the detection of structural abnormalities underlying epileptic seizures, the protocol included T1, FLAIR, T2, SWI, and DWI/ASC, as well as additional sequences such as DIR, T1-weighted C+, and DTI [21].

### 2.3. Neuropsychological Assessment

Neuropsychological assessments were performed in all cases using the Wechsler Adult Intelligence Scale, Fourth Edition [22] and the Wechsler Memory Scale, Fourth Edition [23].

According to the WAIS-IV assessment, 10 subtests were administered to evaluate the Verbal Comprehension Index (Similarities, Vocabulary, Information), Perceptual Reasoning Index (Block Design, Matrix Reasoning, Visual Puzzles), Working Memory Index (Digit Span, Arithmetic), and Processing Speed Index (Symbol Search, Coding).

For the analysis and interpretation of the results, we used subtest scaled scores, composite scores, and discrepancy comparisons. Individual cognitive strengths and weaknesses were also identified.

The Brief Cognitive Status Examination (for adults aged 16–69 years) and 10 core subtests of the WMS-IV were administered to assess five cognitive indexes: Auditory Memory, Visual Memory, Visual Working Memory, Immediate Memory, and Delayed Memory.

For the analysis and interpretation of the results, we calculated index-scaled scores, subtest-level differences within indexes, subtest-level contrast-scaled scores, and index-level contrast-scaled scores. In addition, an Ability–Memory and contrast scaled score analysis was conducted by comparing WAIS-IV and WMS-IV index scores.

### 2.4. Video-EEG Monitoring

Long-term video-EEG monitoring was performed in each patient for a duration of 72 h using a noninvasive protocol (phase Ia) [5] and in accordance with all relevant standards. Interictal and ictal epileptiform features (spike and slow-wave complexes) as well as slowing (rhythmic delta or theta activity) were defined according to the IFCN glossary of terms [24].

Long-term video-EEG recordings were acquired at a sampling rate of 1 kHz using Micromed EEG amplifiers (Mogliano Veneto, Italy). Monitoring was performed in the Epilepsy Monitoring Laboratory of the INN using a 32-channel system (Micromed BQ 3200 ACQDV/LTM32 EXPRESS PCI—32-channel digital video-EEG system; S.p.A., located at Via Giotto 2, 31021 Mogliano Veneto (Treviso), Italy), which is capable of detecting high-frequency oscillations in the range of 80–500 Hz. All technical requirements for vEEG recording were fully met, enabling the capture of both clinical and subclinical events.

During the EEG analysis, interictal and ictal epileptiform features (spike and slow-wave complexes), as well as slowing (rhythmic delta or theta activity), were identified according to the IFCN glossary of terms [24].

From the video-EEG recordings, three different segments of the primary excitatory seizure zone were extracted by a qualified epileptologist/clinical neurophysiologist. At least one electroclinical seizure was visually analyzed for each patient.

According to our concept, the SOZ obtained through mathematical processing of scalp video-EEG monitoring data should correlate with the SOZ identified from seizure semiology, 3T MRI findings, neuropsychological testing, and epilepsy surgery outcomes.

### 2.5. Preprocessing of EEG Recordings

After video-EEG monitoring, an epileptologist extracted six distinct EEG segments from the primary hypothesized SOZ based on visual assessment: interictal, preictal (15, 9, and 6 s before seizure onset), ictal, and postictal periods.

For the analysis of EEG brainwaves, different time windows were selected, specifically 9.5 s, 6.5 s, and 3.0 s segments of preictal spike activity. These segments served as indicators for the initial localization of the seizure onset zone (SOZ).

According to our hypothesis, the SOZ obtained through mathematical processing of scalp video-EEG monitoring data should correlate with the SOZ identified from seizure semiology, 3T MRI findings, neuropsychological testing, and epilepsy surgery outcomes (in cases where surgery was performed).

A well-recognized technical limitation of scalp EEG recordings is the presence of movement-related (muscular) artifacts, which was also observed in our study. These artifacts substantially degrade signal quality and may make accurate interpretation nearly impossible. Modern EEG systems include built-in software tools (e.g., high- and low-pass filters, notch filters) to improve signal quality and facilitate data processing. However, even with advanced filtering methods, it remains impossible to completely eliminate artifacts, since filtering inevitably affects both noise and physiological or pathological activity within overlapping frequency ranges. This considerably complicates the reliable interpretation of EEG data.

For these reasons, the video-EEG recordings were subjected to extensive artifact removal procedures to ensure the highest possible data quality for subsequent mathematical processing, specifically:

(A) EEG recordings were preprocessed using MATLAB (MATLAB and Statistics Toolbox Release 2010b, Update 4, Version 24.1, The MathWorks, Inc., Natick, MA, USA). The EEGLAB toolbox was used to remove evident artifacts and corrupted segments, as well as to attenuate line noise originating from electrical devices [25].
(B) The preprocessed EEG data were further analyzed in EEGLAB using Independent Component Analysis (ICA). This procedure decomposed the signals into independent frequency components, allowing for the separation of neural oscillations from non-neural sources (e.g., muscular activity, line noise, ocular and cardiac artifacts). Non-brain components were removed, after which the EEG data were reconstructed.
(C) In the final step, EEG channels identified as probable seizure onset zones (SOZs) were digitized and prepared for subsequent mathematical processing.

**Figure 1:**
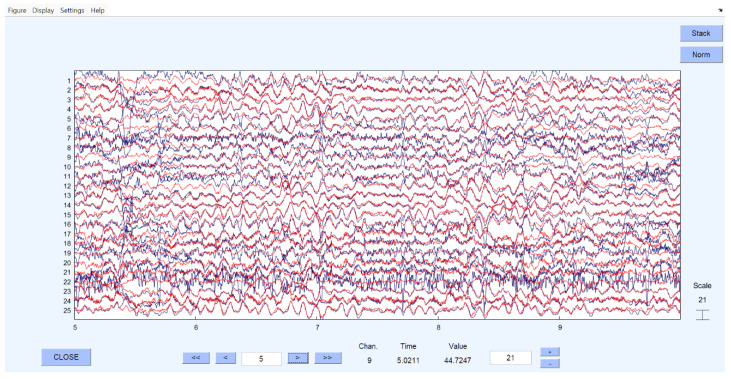
EEG before and after artifact removal (red) by independent component analysis (ICA).

**Figure 2:**
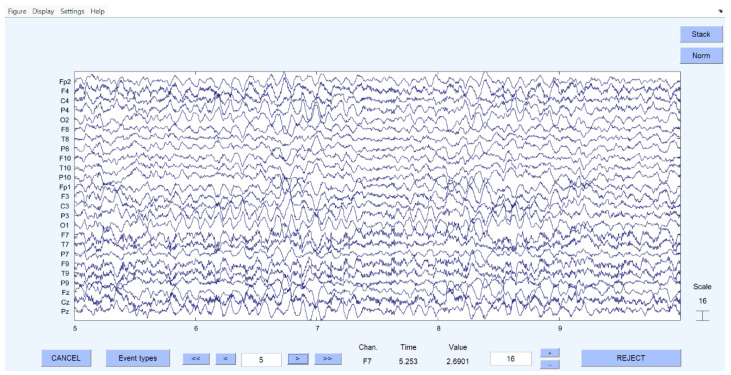
Cleaned EEG after artifact removal by independent component analysis.

Figure 1 and Figure 2 illustrate examples of EEG time series before and after preprocessing in MATLAB/EEGLAB using ICA. Following artifact removal, the EEG data were bandpass-filtered in the 80–500 Hz range to isolate high-frequency oscillations relevant for SOZ identification prior to GC analysis.

In all cases, the regions of interest were selected from either the right or left temporal lobe channels, as all patients demonstrated hippocampal sclerosis on MRI.

### 2.6. Mathematical Justification (Data Analyses Using GC and JLA)

A mathematical validation of the conclusions drawn from visual assessment regarding epilepsy onset zones and their propagation, as discussed in the previous sections, represents one of the main contributions of this study. Granger causality (GC) is one of the leading statistical techniques for inferring the direction of neural interactions and information flow from experimental data [17]. Describing the method in a few words, when one has two simultaneously acquired time series $X_{n}$ and $Y_{n}$, if the autoregressive prediction of the first time series at the present time can be improved by including the past information of the second time series, it is said that Y has a causal influence on X. Expressed quantitatively, if

(1) $$X_{n}=\sum_{k=1}^{\infty }a_{k}X_{n-k}+\varepsilon_{n}$$

and

(2) $$X_{n}=\sum_{k=1}^{\infty }b_{k}X_{n-k}+\sum_{k=1}^{\infty }c_{k}Y_{n-k}+\gamma_{n}$$

are, respectively, the autoregressive and joint representations, then the Granger causality measure is (see Equation (7))

(3) $$I_{X\to Y}=\frac{var(\varepsilon_{n})}{var(\gamma_{n})}.$$

Nonparametric estimation of (1) relies on the factorization of the power spectral density matrix of the stationary stochastic process, as described in a recent paper [26].

(4) $$S\left(t\right)=S_{+}(t)S_{+}^{*}(t)$$

We employ recent developments in GC that take multitime-ahead prediction step (L) into account in causal relations. Specifically, we consider multistep-ahead prediction representations in (1) and (2) and define the L-lag causal relation as

(5) $$I_{X\to Y}^{L}=\frac{var\left(\varepsilon_{n}^{L}\right)}{var(\gamma_{n}^{L})},$$

where

(6) $$\varepsilon_{n}^{L}={inf}_{N,\alpha }\left\|X_{n+L}-\sum_{k=0}^{N}\alpha_{k}X_{n-k}\right\|$$

and

(7) $$\gamma_{n}^{L}={inf}_{N,\alpha ,\beta }\left\|X_{n+L}-\sum_{k=0}^{N}\alpha_{k}X_{n-k}-\sum_{k=0}^{N}\beta_{k}Y_{n-k}\right\|$$

The quantity in (5) measures the proportion of the influence of time series $Y_{n}$ on $X_{n}$, which is distributed within milliseconds, and it can also be obtained from the spectral factorization (4) of the power spectral density (see [26]).

### 2.7. Ethical Issue

The Ethics Committee of the INN (INN-005/2023) approved the project proposal. The study followed the principles outlined in the WMA Declaration of Helsinki. Before enrolling, all study patients agreed to participate in the study and provided written informed consent.

## 3. Results

### Demographics

Six patients with drug-resistant focal epilepsy (aged 28–43 years; three females) who underwent presurgical evaluation using a noninvasive protocol and subsequently received epilepsy surgery were included in the study. Demographic characteristics, epilepsy duration, seizure history, neuropsychological scores, MRI findings, and EEG analyses are summarized in Table 1.

As described in the methodology, selected EEG segments were cleaned, processed, and digitized. Figure 1 and Figure 2 present examples of the EEG data before and after artifact removal and recomposition.

The EEG signal obtained after recomposition of the filtered data is shown in Figure 2, after which digitization and mathematical analysis were performed.

**Figure 3:**
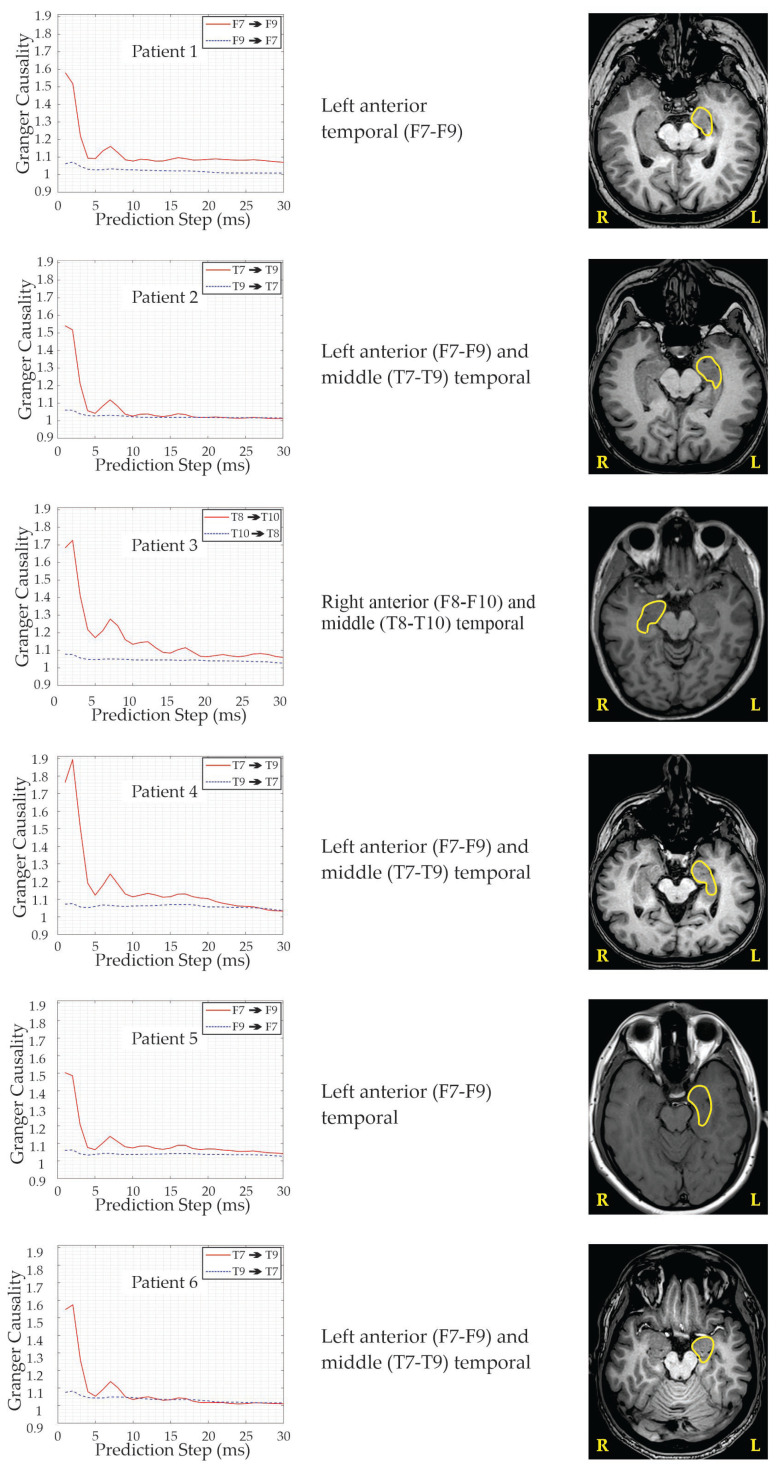
Multistep-ahead Granger causality (GC) estimates (**left**) and corresponding T1-weighted MR images (**right**) of six epilepsy patients. Curves indicate surgically resected regions nearest to the dominant GC electrode identified from ictal video-EEG recordings.

Figure 3 demonstrates a unidirectional spread of excitation in the EEG signals recorded between the corresponding brain regions. For illustration, six representative GC graphs were selected. In all cases, the EEG signals were analyzed over the same time window relative to the reference point, defined as the visually identifiable onset of the seizure.

After examining different options, 1-s EEG segments recorded one second before the reference point were selected for demonstration. The selection of this interval followed the evaluation of numerous segments of varying lengths and distances from the reference point. For each selected interval, the power spectral density matrix was estimated using the multitaper method [17], and spectral factorization (4) was subsequently performed using JLA to compute (5). Since a large number of spectral matrices had to be factorized during this procedure, the JLA was repeatedly applied as part of the nonparametric GC estimation workflow (see Figure 3).

**Figure 4:**
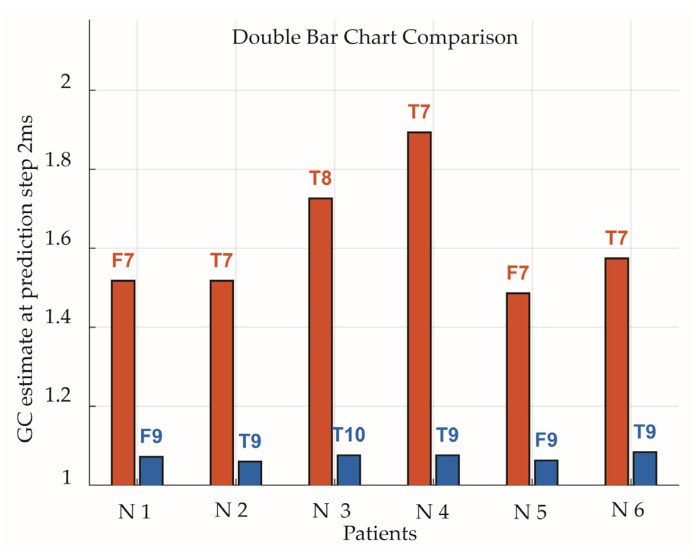
Double-bar chart of Granger causality (GC) estimates at a one-step-ahead prediction horizon (2 ms step) for dominant flow directions (red) and reverse directions (blue). GC values are extracted from the left column of Figure 3.

Because traditional Granger causality (GC) is based on one-step-ahead prediction, GC estimates at the one-step (2 ms) prediction horizon were isolated for comparison and are shown as double-bar charts for the dominant (red) and reverse (blue) directions (see Figure 4).

To provide a comparative reference, pairwise GC analyses also included a small number of contralateral channels (e.g., nonaffected temporal homologues). These regions showed only minimal directional interactions, suggesting that the estimated causal relationships were predominantly confined to the clinically suspected epileptogenic networks. Although preliminary, this observation indicates that the approach did not yield apparent false-positive directional connections in these representative cases.

## 4. Discussion

In the present study, the JLA was applied for the first time to process real scalp EEG data from six patients with drug-resistant epilepsy. Specifically, the JLA was utilized to estimate LLL-lag-ahead GC within a nonparametric framework. The preliminary findings demonstrated that, in all six surgically treated patients, the seizure onset zone (SOZ) and propagation patterns identified through standard scalp video-EEG modalities were consistent with those obtained using this mathematical approach.

Drug-resistant epilepsy is associated with significant psychosocial, behavioral, medical, and economic consequences. Surgical intervention should be considered in a timely manner for patients with drug-resistant focal epilepsy [27]. Given that seizure freedom following epilepsy surgery can be achieved in 60–80% of individuals with focal epilepsy, any approach that contributes to improved SOZ identification—particularly in extra-temporal or multifocal epilepsies—may play an important role in enhancing diagnostic accuracy, surgical outcomes, and overall quality of life for these patients.

**Figure 5:**
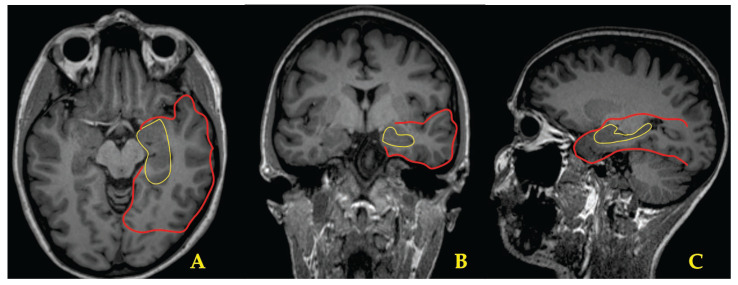
Schematic representation of the temporal lobe (red line) and hippocampal anatomy (yellow line) on axial (**A**), coronal (**B**), and sagittal (**C**) T1-weighted MRI images.

Accurate presurgical evaluation and precise localization of the SOZ and its propagation pathway—based primarily on ictal/interictal scalp video-EEG monitoring, epilepsy-protocol MRI, and neuropsychological testing—are critical for achieving successful surgery and long-term seizure freedom [28]. To facilitate understanding of the region evaluated for potential surgical intervention in our patients, Figure 5 highlights the relevant anatomical areas.

However, these noninvasive modalities are not always sufficient, and many patients require prolonged invasive video-EEG monitoring with intracranial electrodes, strips, or grids, along with additional neuroimaging studies [29]. Such procedures significantly increase diagnostic costs and, in addition to being relatively invasive, may ultimately prove uninformative, potentially exacerbating the patient’s psychosocial burden.

Although intracranial EEG (iEEG) studies are an effective method for identifying the primary epileptogenic focus, particularly in cases involving extratemporal, large, deep, or multiple foci [30], invasive monitoring carries risks of several adverse events, including infection, hemorrhage, and elevated intracranial pressure [31,32,33].

To avoid such problems, high-frequency oscillations (HFOs) have recently been actively used as biomarkers of epileptogenicity. They have been shown to originate from small, localized brain regions and can, in some cases, be recorded from the scalp [34,35]. Although scalp EEG has limited spatial resolution due to volume conduction, GC-based directional analysis may partially mitigate this limitation. Future research may further refine SOZ estimation by incorporating source localization methods (e.g., sLORETA) prior to GC computation.

For this reason, the analysis of high-frequency (>80 Hz) oscillations using nonparametric GC has been reported to be successful [6]. GC is an autoregressive statistical method [14] based on the theory of stochastic processes [36] that calculates predictions from time series data and is widely used to analyze continuous neural signals. The concept of GC can be summarized as follows: if the current value of one time series can be better predicted by incorporating past values of a second time series, then the second time series is said to have a causal influence on the first.

Because long-term video-EEG monitoring reflects continuous neuronal activity, the GC framework can be naturally adapted to EEG analysis. This approach has been effectively applied to the assessment of seizure onset zone (SOZs) in patients with drug-resistant epilepsy, as demonstrated by Adhikari and colleagues [6]. GC, used in a nonparametric form for high-frequency oscillations (>80 Hz), has shown encouraging results in the analysis of seizure propagation and epileptogenicity [6].

To compute nonparametric GC, matrix spectral factorization is required. In neuroscience research, Wilson’s algorithm (WA) has traditionally been the most widely used method for this purpose [16]. More recently, an alternative approach, the Janashia–Lagvilava algorithm (JLA) [18], has also been explored in mathematical and engineering studies [19,20,37]. Although originally developed outside the neuroscientific context, JLA has shown reliability in noisy environments, maintaining stable variance in synthetic datasets even with added Gaussian noise [19,37]. Its mathematical properties suggest that it may serve as an additional tool for GC computation in clinical research, particularly when working with noninvasive scalp EEG.

Unlike prior work in SOZ localization, which has mainly relied on single-lag or frequency-restricted GC applied to invasive iEEG recordings (e.g., [6,17]), the present study applies multi-lag GC computations to noninvasive scalp EEG. This was made possible by stable spectral factorization using the JLA. Multi-lag analysis allows temporal dynamics of ictal information flow to be examined across multiple millisecond-level prediction steps, providing a complementary, mathematically grounded perspective to standard presurgical evaluation.

In this preliminary study, we applied JLA-based nonparametric GC to scalp EEG recordings in six patients with drug-resistant focal epilepsy and compared the results with standard presurgical assessments. In all cases that subsequently underwent surgery, the SOZ and propagation patterns identified using conventional clinical modalities were consistent with those obtained using this mathematical approach. These initial observations indicate that JLA-based GC may serve as a feasible supplementary modality for presurgical evaluation under noninvasive protocols. While this possibility is particularly compelling given the temporal instability of network interactions in epilepsy, further validation in larger cohorts is necessary to assess robustness and clinical applicability.

## 5. Strengths and Limitations

The primary strength of this study lies in the application of a well-established mathematical framework—the JLA—within clinical epileptology for the first time. By integrating this method into nonparametric Granger causality analysis, we demonstrated its feasibility for assessing seizure onset zones using scalp vEEG recordings under a noninvasive presurgical protocol, alongside seizure semiology, MRI findings, and neuropsychological evaluation. Rather than replacing existing methods, this approach provides mathematical support for visual interpretation of ictal EEG patterns and illustrates that advanced spectral factorization techniques may be meaningfully incorporated into presurgical assessment. Overall, these findings highlight the broader potential for mathematical algorithms to contribute to clinical decision-making in neuroscience.

Nevertheless, several limitations should be acknowledged. First, the study involved a small cohort (*n* = 6), which limits generalizability and requires validation in larger populations with diverse epileptic etiologies. Second, only time domain, multi-lag GC was applied. Frequency-resolved GC—which could provide additional insight into oscillatory interactions within specific HFO ranges (e.g., 80–250 Hz ripples [7])—was not implemented in the present analysis. Incorporating band-specific GC and a broader patient sample, therefore, represents an important direction for future research, with the potential to clarify the clinical utility of this approach in presurgical evaluation.

## 6. Conclusions

The application of JLA-based spectral factorization to noninvasive EEG illustrates that this mathematical framework can be incorporated into presurgical analysis of directional neural interactions. By integrating advanced spectral factorization with clinical EEG evaluation, the approach demonstrates a feasible link between theoretical signal processing and practical clinical assessment. Rather than serving as a replacement for invasive methods, the JLA-based nonparametric GC framework offers a supplementary perspective that may contribute to decision-making in complex presurgical cases. Beyond clinical use, this methodology provides a scalable tool for studying directional information flow in neural systems, with potential relevance to neuroengineering, brain–computer interfaces, and data-driven neuromodulation strategies. Future integration with frequency-dependent GC could further refine the study of HFO-related biomarkers and broaden the framework’s applicability.

## Figures and Tables

**Table 1:** Demographic and clinical characteristics of study participants.

Patient’s N/Age/Gender	Age at Seizure Onset	Seizure Semiology	EEG Interictal	EEG Ictal with Scalp Electrode Placement	MRI Eloquent Area/Side	Neuro-Psychology/Area/Side	Concordance of the Modalities	Side and Area of Performed Surgery	Early Post-Surgery Period	Outcome	Follow Up (Months)
1/28/Male	6	Focal—with fear, impaired consciousness and oral automatisms	Bi-Temporal	L-anterior Temporal (F7–F9)	L-HS	L-Medial Temporal	+(−)+++	L-Hippo-campectomy (AASJ15)	One FBTCS 3rd month after surgery	Engel 1a	16
2/40/Female	20	Focal—preserved consciousness, with pressure in head and heating	L-Temporal	L-anterior (F7–F9) and middle (T7–T9) Temporal	L-HS	Bi-Frontal, R-Parietal	++++(−)	L-Hippo-campectomy (AASJ15)	_	Engel 1b	5
3/42/Female	9	Focal—unpleasant sensation in the mesogastrium, with fear and impaired consciousness	Bi-Temporal	R-anterior (F8–F10) and middle (T8–T10) Temporal	R-HS	Bi-temporal	+(−)++(−)	R-Hippo-campectomy (AASJ15)	_	Engel 1a	9
4/43/Female	27	Focal—epigastric aura, tachycardia, impaired consciousness and oral automatisms	L-Temporal	L-anterior (F7–F9) and middle (T7–T9) Temporal	L-HS	Bi-Temporal	++++(−)	L-Hippo-campectomy (AASJ15)	One FBTCS within 24 h of surgery	Engel 1a	9
5/43/Male	14	Focal—with unpleasant taste, “uncooked beans”, tachycardia, inadequate speech; Impairment of consciousness, oral and manual automatisms, more prominent in the left hand.	L-Temporal	L-anterior Temporal (F7–F9)	L-VS	R-Temporal and Frontal	++++(−)	L-Hippo-campectomy (AASJ15)	One FBTCS within 24 h of surgery	Engel 1a	5
6/30/Male	8	Feeling of ringing in the ears, unexplained fear, impaired consciousness, deviation of the head and eyes to the left, oral and left manual automatisms, dystonia in the right hand, up to 1 min	L-Temporal	L-anterior (F7–F9) and middle (T7–T9) Temporal	L-HS L-Peri-ventricular Heterotopia	L-Temporal	+++++	L-Hippo-campectomy (AASJ15)	_	Engel 1a	5

Abbreviators: HS, hippocampal scleroses; L, left side; R, right side; Bi, bilateral. FBTCS, focal bilateral tonic–clonic seizures.

## Data Availability

Data will be made available upon reasonable request. The dataset is not publicly accessible due to privacy and ethical restrictions.
